# Marginal vitamin C status is associated with reduced fat oxidation during submaximal exercise in young adults

**DOI:** 10.1186/1743-7075-3-35

**Published:** 2006-08-31

**Authors:** Carol S Johnston, Corinne Corte, Pamela D Swan

**Affiliations:** 1 Department of Nutrition, Arizona State University, Mesa, USA; 2 Department of Exercise and Wellness, Arizona State University, Mesa, USA

## Abstract

**Background:**

Vitamin C is a cofactor in the biosynthesis of carnitine, a molecule required for the oxidation of fatty acids. A reduction in the ability to oxidize fat may contribute to the reported inverse relationship between vitamin C status and adiposity. To examine this possibility, we conducted a preliminary trial to evaluate the impact of vitamin C status on fat oxidation during submaximal exercise.

**Methods:**

Fat energy expenditure was determined in individuals with marginal (n = 15) or adequate (n = 7) vitamin C status during a submaximal, 60-minute treadmill test. Subsequently, eight of the subjects with marginal vitamin C status completed an 8-week double-blind, placebo-controlled, depletion-repletion trial with submaximal exercise testing.

**Results:**

Individuals with marginal vitamin C status oxidized 25% less fat per kg body weight during the treadmill test as compared to individuals with adequate vitamin C status. Fat oxidation during exercise was inversely related to fatigue (r = -0.611, p = 0.009). Vitamin C repletion of vitamin C depleted subjects (500 mg vitamin C/d) raised fat energy expenditure during exercise 4-fold as compared to depleted control subjects (p = 0.011).

**Conclusion:**

These preliminary results show that low vitamin C status is associated with reduced fat oxidation during submaximal exercise. Low vitamin C status may partially explain the inverse relationship between vitamin C status and adiposity and why some individuals are unsuccessful in their weight loss attempts.

## Background

About 15% of US adults are vitamin C deficient (plasma vitamin C < 11 μmol/L) [1]. Twenty-five years ago, this percentage was much lower, 3–5% of US adults [2]. Concomitant with this recent rise in the prevalence of vitamin C deficiency, America experienced a major epidemic of overweight and obesity [3]. Indeed, survey data suggest an inverse relationship between vitamin C status and body weight [4,5] and waist measurements [6]. The underlying systemic oxidative stress associated with obesity has been proposed to explain the inverse relation between adiposity and plasma vitamin C concentrations [6]. Since vitamin C is an essential cofactor for the biosynthesis of carnitine, a molecule required for the oxidation of fatty acids [7,8], a reduction in the ability to oxidize fat may contribute to the relationship between vitamin C status and adiposity. To examine this possibility, we conducted a preliminary trial to evaluate the impact of vitamin C nutriture on fat oxidation during submaximal exercise in a small sample of young adults.

## Methods

Seventy-eight sedentary, non-smoking men and women, aged 18–38 y, from a campus population were screened for plasma vitamin C status. Nearly 40% of the sample had marginal vitamin C status (n = 30; plasma vitamin C concentration <34 μmol/L), and 15 of these individuals (mean age, 21.1 ± 0.8 y) agreed to participate in exercise testing. An additional 7 of the screened individuals with adequate vitamin C status (plasma vitamin C concentration ≥ 34 μmol/L) were included as the vitamin C adequate (control) group. All participants provided informed consent, and the study was conducted in accordance with the guidelines of the University Institutional Review Board.

Participants were free of medical conditions contraindicated for exercise testing as set by guidelines of the American College of Sports Medicine. Participants did not regularly use nutritional supplements and were unaware of their vitamin C status or the intent of the study. Maximal oxygen consumption (VO_2 _max) using a graded walking protocol was determined. Within 7–10 days of VO_2 _max testing, participants returned to the laboratory for a submaximal, 60-minute treadmill walk at 50% of their VO_2 _max. Participants were instructed to fast for 4–6 h prior to the exercise test and to remain sedentary for the previous 24 h period. Urine samples were collected pre- and post-exercise to assess urea nitrogen. During the exercise test, oxygen consumption and respiratory exchange ratio (RER) were measured from three minute gas samples collected at 10 minute intervals corresponding to minutes 7–10, 17–20, 27–30, etc. Substrate utilization was then calculated using the non-protein respiratory quotient derived from the urinary nitrogen and metabolic (VO_2 _and VCO_2_) measures. The data reported represent averaged values from samples collected at steady state toward the end of exercise (i.e., 37, 47, and 57 minutes). Immediately after testing, participants completed the POMS questionnaire (Profile of Moods States; EdiTS/Educational and Industrial Testing Service, San Diego, CA).

### Intervention trial

Eight of the 15 participants with marginal vitamin C status agreed to participate in an 8-week intervention trial. These subjects maintained their usual dietary patterns; however, they were provided with a list of foods to avoid during the trial. [Although not revealed to the subjects, these foods contained >30 mg of vitamin C per serving.] During weeks 1–4, all participants ingested a placebo capsule daily. At the end of this depletion period, subjects completed a submaximal, 60-minute treadmill walk as described above. Starting at week 5, the participants were randomly assigned to receive a placebo capsule (depleted group; n = 3) or a 500 mg vitamin C capsule (repleted group; n = 5) daily. A double-blind protocol was followed, and the placebo and vitamin C capsules were identical in appearance. At the end of week 8, participants completed a 60-minute treadmill walk. Within one week of the completion of the trial, participants completed a second VO_2 _max test utilizing the procedures outlined above. Fasting blood samples were collected at weeks 5 and 8. An aliquot of plasma was deproteinized in 10% TCA and frozen at -45°C until analyzed for vitamin C using 2,4-dinitrophenylhydrazine [9]. Plasma free carnitine was measured radiochemically [10], and urinary urea nitrogen was measured colorimeterically.

Data are reported as mean ± SE, and statistical analyses were performed using SPSS for WINDOWS (version 12; SPSS Inc, Chicago). Differences between means were assessed using an independent t-test, and the Pearson's correlation was used to identify relationships between variables. Significance was set at p ≤ 0.05.

## Results

Data from the descriptive study showed that RER during submaximal exercise was significantly higher for individuals with marginal vitamin C status as compared to the vitamin C adequate controls (p = 0.034; Table 1). Furthermore, fat oxidation during exercise was reduced 25% for the marginal versus adequate vitamin C groups (p = 0.045). Plasma free carnitine was significantly lower in the controls as compared to the subjects with marginal vitamin C status (Table 1). (Although carnitine concentrations in muscle are directly related to vitamin C status, plasma carnitine concentrations appear to be inversely related to vitamin C status [11].) Fat oxidation during the walking test was inversely correlated to fatigue as scored by the POMS questionnaire (r = -0.611, p = 0.009) and to plasma carnitine (r = -0.489, p = 0.034). Plasma vitamin C was inversely related to plasma carnitine (r = -0.794, p = 0.000), but the correlation between plasma vitamin C and fat oxidation or RER did not achieve significance (r = 0.309 and r = -0.329 respectively).

### Intervention trial

**Figure 1:**
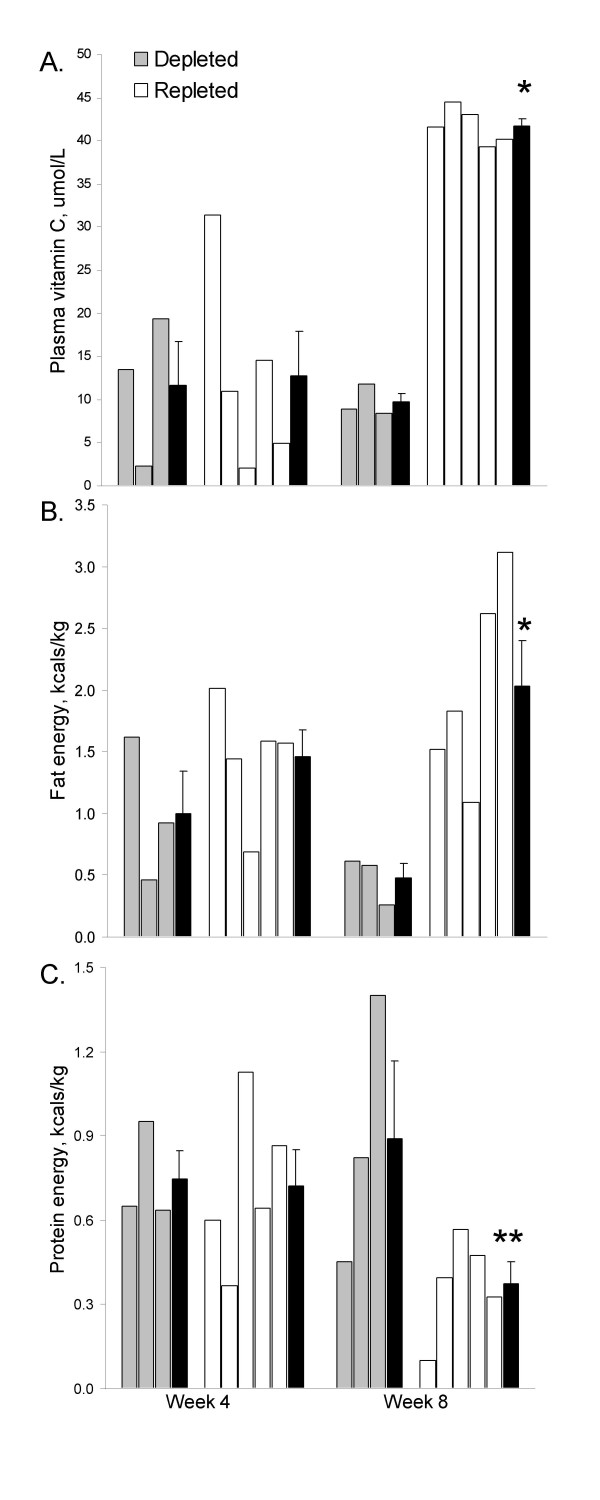
Individual subject data for plasma vitamin C concentration (A) and for fat energy (B) and protein energy (C) expended during submaximal exercise in vitamin C depleted (placebo capsule daily; n = 3) and vitamin C repleted (500 mg vitamin C capsule daily; n = 5) subjects at week 4 (pre-intervention) and week 8 (post-intervention). Means (± SE) are shown in black bars; *p < 0.05; **p = 0.062 (independent t-test)

Plasma vitamin C concentrations after the depletion period (weeks 1–4) averaged 12.3 ± 3.5 μmol/L. Following the randomized intervention period (weeks 5–8), plasma vitamin C concentrations differed significantly between the repleted and depleted groups (41.7 ± 0.9 and 9.7 ± 1.0 μmol/L respectively, p < 0.001; Figure 1). Fat oxidation was also significantly different by group at week 8 (2.03 ± 0.37 and 0.48 ± 0.11 kcals/kg for the repleted and depleted groups respectively, p = 0.011; Figure 1), and protein oxidation was elevated at week 8 in the depleted group as compared to the repleted group (0.89 ± 0.28 and 0.37 ± 0.08 kcals/kg respectively, p = 0.062).

When week 4 and week 8 data were combined, plasma vitamin C concentrations were inversely related to protein energy expenditure (r = -0.679, p = 0.004) and directly related to fat energy expenditure (r = 0.655, p = 0.006). Plasma carnitine was weakly related to plasma vitamin C (r = -0.441, p = 0.087). A training effect was not evident in participants since pre- and post-trial VO_2 _max values were similar (34.3 ± 3.7 and 36.7 ± 7.9 ml/kg/min versus 32.9 ± 4.1 and 34.9 ± 6.7 ml/kg/min for the repleted and depleted groups respectively at pre-trial versus post-trial).

## Discussion

These preliminary data indicate that vitamin C status impacts fat oxidation. Free-living individuals with marginal vitamin C status oxidized 25% less fat per kg body weight during a 60-minute treadmill walk as compared to individuals with adequate vitamin C status. Moreover, fat oxidation during exercise was enhanced in these individuals by normalizing plasma vitamin C concentrations. Vitamin C is a cofactor for two enzymes required for the biosynthesis of carnitine, ε-*N*-trimethyl-L-lysine hydroxylase and γ-butyrobetaine hydroxylase [12-15]. Since the oxidation of fatty acids in skeletal muscle is dependent on carnitine [7,8], this is a possible mechanism by which vitamin C affects fat oxidation. We also observed a reduction in protein oxidation in the vitamin C repleted subjects which is consistent with the protein-sparing effect of supplemental carnitine [16].

Numerous trials have demonstrated that tissue carnitine levels are directly related to vitamin C nutriture [11,17,18], and that plasma vitamin C and carnitine concentrations are inversely related [11,19]. However, to our knowledge, only one other study has examined the relationship between vitamin C, carnitine metabolism, and fat oxidation [20]. In cultured hepatocytes, Ha et al. demonstrated that ascorbic acid directly stimulated carnitine synthesis and the β-oxidation of fatty acids, and reduced triglyceride accumulation [20]. This observation that vitamin C reduces fat deposition may explain the reported inverse relationship between adiposity and vitamin C status [4-6]. Interestingly, in patients with an inborn error of carnitine metabolism, carnitine deficiency is associated with lipid accumulation in tissues [21-23]. In fact, activation of the carnitine system has been cited as a possible treatment for obesity [24-26].

Carnitine deficiency is also associated with fatigue and exercise intolerance [21-23], and Hughes et al postulated several decades ago that the lassitude and tiredness of scurvy may be attributed to carnitine deficiency [17]. Our data indicated that reduced fat oxidation during exercise was related to fatigue. It is tempting to speculate that the reduced fat oxidation associated with vitamin C depletion may result in weight gain by two mechanisms: indirectly by fatigability and exercise intolerance and directly by lipid accumulation. Since 15% of Americans are reportedly vitamin C deficient, and one-third of Americans have below adequate vitamin C status [1,27], these issues deserve further investigation.

Our study had several limitations. The sample sizes were small, 22 for the descriptive study and eight for the intervention trial, which limits the generalizability of results. Furthermore, muscle carnitine, the most sensitive marker of tissue carnitine status, was not assessed in this trial. Also, since vitamin C is a required cofactor for dopamine beta-hydroxylase [28], altered norepinephrine responses in vivo may have impacted fat oxidation in this trial [see [29]].

## Conclusion

These preliminary results show that low vitamin C status may reduce fat oxidation during submaximal exercise and that reduced fat oxidation during exercise was related to fatigue. It is possible that increased fatigue and less reliance on fat as a fuel during activity may influence eventual weight gain. Thus, in addition to emphasizing calorie control and physical activity, attention to specific diet components such as vitamin C may be necessary for effective weight management.

## Abbreviations

RER: respiratory exchange ratio

**Table 1:** Baseline characteristics for subjects with marginal (n = 15) or adequate (n = 7) vitamin C status

	Marginal vitamin C status Plasma vitamin C <34 μmol/L	Adequate vitamin C status Plasma vitamin C ≥34 μmol/L	p value^1^
Vitamin C, μmol/L	18.1 ± 2.5	42.9 ± 3.4	.000
BMI, kg/m^2^	25.0 ± 1.3	23.3 ± 1.4	.436
Total carnitine, ng/ml	12.0 ± 0.8	6.5 ± 0.9	.000
RER^2^	0.87 ± 0.02	0.83 ± 0.01	.034
Fat energy*, kcals/kg	1.02 ± 0.12	1.36 ± 0.11	.045
VO2max*, ml/kg/min	37.5 ± 2.5	36.9 ± 2.3	.866

^1^Independent t-test.

^2^Computed at steady state during 60-minute submaximal walk test.

## Competing interests

The author(s) declare that they have no competing interests.
